# The phosphoproteome of ovarian carcinoma delineates signaling signatures and potentially druggable targets across histotype stages

**DOI:** 10.1038/s41698-026-01659-9

**Published:** 2026-08-14

**Authors:** Lucas Werner, Parisa Esmaeili, Ella Ittner, Hugo Swenson, Anikó Kovács, Claudia Mateoiu, Pernilla Dahm-Kähler, Per Karlsson, Toshima Parris, Fredrik Levander, Khalil Helou

**Affiliations:** 1 Department of Oncology, Institute of Clinical Sciences, Sahlgrenska Academy, University of Gothenburg, Gothenburg, Sweden; 2 Sahlgrenska Center for Cancer Research, Sahlgrenska Academy, University of Gothenburg, Gothenburg, Sweden; 3 Department of Immunotechnology, Science for Life Laboratory, Lund University, Lund, Sweden; 4 Department of Clinical Pathology, Region Västra Götaland, Sahlgrenska University Hospital, Gothenburg, Sweden; 5 Institute of Biomedicine, Sahlgrenska Academy, University of Gothenburg, Gothenburg, Sweden; 6 Department of Obstetrics and Gynecology, Institute of Clinical Sciences, Sahlgrenska Academy, University of Gothenburg, Gothenburg, Sweden; 7 Department of Oncology, Region Västra Götaland, Sahlgrenska University Hospital, Gothenburg, Sweden

**Keywords:** Cancer, Cell biology, Oncology

## Abstract

Ovarian malignancy is the most lethal gynecologic tumor, with an 80% relapse rate and 47% five-year survival. Epithelial ovarian cancer (EOC), the most common form, comprises five major histotypes with distinct clinicopathological characteristics, yet treatment still relies largely on cytoreductive surgery and platinum-based chemotherapy. Phosphoproteomics enables the mapping of protein phosphorylation, a key hallmark of cellular signaling. This study aimed to define phosphoproteomic-based ovarian cancer subgroups and assess their relationships with pathway activation patterns, patient prognosis, and therapeutic relevance. We analyzed the phosphoproteome of four major EOC histotypes from 180 patients. Dysregulated phosphosites critical for carcinogenesis were identified, including NUCKS1:S181, WWTR1:S89, and SRRM2:T1880. Kinase activity revealed histotype-specific CDK1/2 signatures linked to EOC-related signaling pathways. Prognostic phosphosites displayed stage- and histotype-dependent patterns. Collectively, these findings elucidate signaling cascades driving disease progression and identify potential therapeutic targets in early and advanced EOC, supporting future efforts toward the development of histotype-specific treatment strategies.

## Introduction

Ovarian carcinoma is the eighth most common malignancy among women and the deadliest gynecologic malignant tumor, accounting for over 200,000 deaths worldwide in 2020^1,2^. The majority of cases (90%) originate from the epithelial layer of the ovaries or fallopian tubes and are classified as epithelial ovarian cancer (EOC)^1^. According to the 2020 World Health Organization (WHO) guidelines, EOC comprises five major histotypes, namely high-grade serous ovarian carcinoma (HGSC), low-grade serous ovarian carcinoma (LGSC), endometrioid ovarian carcinoma (EC), mucinous ovarian carcinoma (MC), and clear cell ovarian carcinoma (CCC)^3^. EOC is typically asymptomatic in early stages (I or II), whereas advanced stage disease (III or IV) often presents with nonspecific symptoms such as abdominal pain, menstrual irregularities, and bloating^4,5^. Additionally, clinical tools for early and reliable detection and treatment remain limited. The widely used biomarker CA-125 is elevated in only 50% of stage I cases, and may increase due to other malignancies or hormonal changes^4,6^. Collectively, this likely contributes to approximately 70% of patients being diagnosed at an advanced stage^7^.

For the last four decades, cytoreductive surgery followed by platinum-based chemotherapy have been the standard of care for EOC regardless of histotype, despite distinct molecular and clinicopathological differences between these subtypes^8,9^. Although initial response is good, about 75% of patients with advanced disease relapse^10^. MC and CCC respond poorly to chemotherapy, while HGSC is chemo-sensitive and can respond after multiple relapses. However, most patients eventually obtain resistant disease^11^. Consequently, long-term survival (> 5 years) is poor (47% overall, 29% for advanced stage disease) and has seen minimal improvement over the last 30 years (39% to 45% between 1983 and 2012)^9,12^. Therefore, there is a need for histotype-specific biomarkers to improve clinical decision making.

Although EOC has historically been studied as a single disease entity, gene- and protein expression profiling have identified histotype-specific biomarker candidates such as MUC5AC for MC, Napsin A and HNF1B for CCC as diagnostic tools, and genomic analysis has resulted in the development of PARP-inhibitors for BRCA1/2 mutation carriers targeting HRD deficiencies for maintenance therapy, a deficiency predominantly seen in HGSC patients^13–17^. Additionally, folate receptor alpha (FRα) has revealed to be correlated with HGSC histology, and may be a suitable target for platinum-resistant disease^18^. Immunotherapy has shown low efficacy in histotypes, although targeting PD-1/PD-L1 has shown moderate effect in CCC^10^. Although histotype-independent, HER2 overexpression has shown to predict poor survival^19^. Overall, except for targeting the HR deficiencies in HGSC by using PARP-inhibitors, there is no clinically established treatment regimen that exploit the molecular profiles of the histotypes to our knowledge. As such, this necessitates increasing our biological understanding of the underlying molecular attributes of the histotypes and use this information to develop targeted therapies, as studies have shown that molecular profiling can identify subsets of patients susceptible and resistant to therapies as well as novel therapeutic targets^20^.

Phosphoproteomics maps the phosphorylation landscape of proteins and is an additional omics layer that can provide novel biomarker candidates^21^. Given that protein activity is regulated by kinase-mediated phosphorylation of serine, threonine, and tyrosine, which are key post-translational modifications (PTMs) governing cell signaling, phosphoproteomics enables the identification of aberrantly phosphorylated sites and dysregulated kinases. These alterations underlie cancer development and progression, and provide targets for treatments^22,23^. Compared with genomics, transcriptomics, or proteomics alone, phosphoproteomics provides additional functional insight into protein activation states and dynamic signaling pathway regulation. In-depth phosphorylation status cannot be to monitored using standard proteomics or other omics approaches; therefore, mass spectrometry phosphoproteomics can provide targets can contribute to the development of precision medicine strategies as well as the discovery of signaling pathways driving carcinogenesis^24^. A previous phosphoproteomics study by Olsen et al. identified CDK7 as a potential druggable target in EOC^25^. Further studies on the ovarian phosphoproteome have identified constituents in the MAPK pathway, PDGFR, and ErbB2-specific phosphoproteins as potential targets^26–28^. Still, however, the phosphoproteome of EOC remains poorly characterized across the major histotypes.

In this study, we profiled the phosphoproteome of primary tumors from 180 EOC patients diagnosed with HGSC, EC, MC, and CCC across all stages (I-IV). By stratifying samples into early stage (I-II) and advanced stage (III-IV) groups, we aimed to identify histotype- and stage-specific phosphosite signatures and signaling pathways using mass spectrometry to provide targets of potential clinical utility. Our findings highlight dysregulated phosphosites and kinases that warrant further validation for prognostic and therapeutic utility. Furthermore, the mapping of cell signaling provides insight into histotype-specific key regulatory disease mechanisms at a previously unexplored level, supporting the development of targeted therapies. Collectively, this study provides a resource understanding histotype-specific signaling events in EOC and highlights candidate phosphosites and pathways for future clinical investigation.

## Results

### Phosphoproteomic profiling of EOC patient cohort

The cohort comprised 180 patients diagnosed with primary EOC, stratified into an early-stage (*n* = 77; 37 HGSC, 20 EC, 8 MC, and 12 CCC cases) and advanced stage group (*n* = 103; 30 HGSC, 19 EC, 25 MC, and 29 CCC cases). A more detailed description of the cohort can be found in Table 1. By performing liquid chromatography-mass spectrometry on phosphopeptide enriched samples from fresh-frozen primary tumor tissues, we identified 8352 unique phosphosites of high localization probability (≥ 0.75) and precursor confidence (*q*-values ≤ 0.01) from 3652 proteins. Proteins were primarily phosphorylated on the Serine amino acid (88%, Supplementary Fig. 1). After filtering for phosphosites detected in at least 30% of the samples in at least one histotype stage group (I-II or III-IV), 4023 sites were retained for further downstream analysis.

**Table 1:** Cohort design and clinicopathologic data after histotype reclassification

	HGSC	EC	MC	CCC
Total samples	67	39	33	41
Patient age				
Mean	63	61	60	61
Range	38–86	25–86	30–86	40–84
Cause of death				
Ovarian carcinoma	40	15	18	20
Other	17	11	12	16
Alive	10	13	3	5
Stage				
I	22	14	7	10
II	15	6	1	2
III	25	15	20	24
IV	5	4	5	5
Dualistic model				
Type I	0	39	33	41
Type II	67	0	0	0

*CCC* Clear cell ovarian carcinoma, *EC* Endometrioid ovarian carcinoma, *HGSC* High-grade serous ovarian carcinoma, *MC* Mucinous ovarian carcinoma.

### Differentially phosphorylated sites reveal stage-specific dysregulation between histotypes

**Fig. 1:**
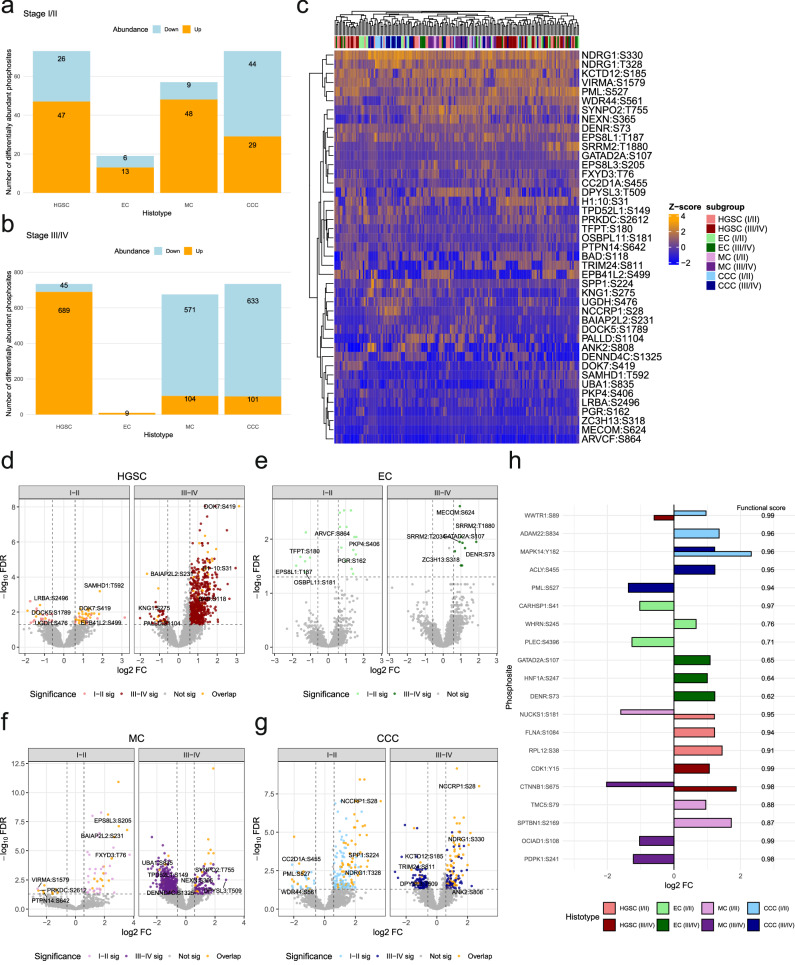
Phosphoproteomic landscape of the EOC histotypes. Bar plot displaying the number of differentially phosphorylated sites with directionality (up- or downregulated) when intensity for a histotype was compared to the others combined for **a** early stages (I/II) and **b** advanced stages (III/IV). **c** Hierarchical clustering heatmap of stage-stratified histotypes using the top 3 significantly up- and downregulated (FDR ≤ 0.05, | FC | ≥ 1.5) sites based on log2 FC for each histotype and stage group. Volcano plots projecting the significantly dysregulated phosphosites with labels for sites displaying the highest and lowest FC, with orange color indicating overlap between early- and advanced stages for **d** HGSC, **e** EC, **f** MC, and **g** CCC. **h** Barplot of ranked phosphosites in both stage groups for each histotype based on estimated functional scores of differentially abundant sites. CCC Clear cell ovarian carcinoma, EC Endometrioid ovarian carcinoma, FDR False discovery rate, HGSC High-grade serous ovarian carcinoma, MC Mucinous ovarian carcinoma, log 2 FC log2 fold change. FDR is based on *p*-values from two-sided empirical Bayes moderated t-tests.

To identify histotype-specific phosphorylation in early- and advanced stage disease, phosphosite abundances were examined to derive signatures based on phosphorylations distinct for a given histotype in early- and advanced stage when using the other histotypes as baseline. A principal component analysis (PCA) revealed a high degree of overlap in abundance between all histotypes in both stage groups (Supplementary Fig. 2). Here, we compare the average intensities of phosphosites in one histotype with the average of all other histotypes combined within the stage groups. In the differential phosphorylation analysis, significantly dysregulated (Benjamini-Hochberg FDR ≤ 0.05, | FC | ≥ 1.5) sites were identified for HGSC, EC, MC, and CCC in both early- and advanced stage comparisons (Fig. 1a, b), though few were detected for EC where no downregulated sites were identified in advanced stage. Notably, the phosphosites tended to be upregulated in HGSC and downregulated in MC and CCC in advanced stage disease. In early stages, such clear distinctions could not be observed. Hierarchical clustering of significant phosphosites of highest and lowest fold change (FC) for each histotype resulted in more distinct grouping of histotypes relative to PCA, except for EC samples and early-stage HGSC (Fig. 1c, Supplementary Fig. 2).

An inspection of differentially phosphorylated sites revealed distinct signatures for each histotype in both stage groups. Phosphorylation at DOK7:S419 (HGSC) and NCCRP1:S28 (CCC) displayed stage-independent upregulation. In EC, only stage-dependent dysregulation was observed, where PKP4:S406 and SRRM2:T1880 were highly upregulated in early- and advanced disease, respectively (Fig. 1d-g, Supplementary Data 1). A list of the most up- and downregulated sites in early- and advanced stages are presented in Table 2, and complementary lists of differentially phosphorylated sites where intensities have been adjusted by protein abundance from a proteomics dataset from the same cohort^29^ prior to analysis can be found in Supplementary Table 1 and Supplementary Data 2. The parental proteins of the dysregulated sites were also differentially abundant in the proteomics dataset to a varying extent between histotype stage groups (FDR ≤ 0.05, | FC | ≥ 1.5 for both datasets). Largest overlap could be seen for early-stage MC (47% of phosphosites), while smallest overlap was found for advanced-stage HGSC (5%) beyond EC that presented no overlap in any stage group. For all observed overlaps, the regulation of phosphosites and proteins was in the same direction, except for CP:S722 and CDCA3:S87 that were upregulated on protein level but downregulated on phosphosite level in advanced-stage MC (Supplementary Data 3).

**Table 2:** Phosphosites of highest differential abundance for each histotype

Histotype	Stage	Protein accession	Phosphosite	FDR	Log2 FC
Upregulated					
HGSC	I/II	O43491	EPB41L2:S499	2.06e-2	3.15
	III/IV	Q18PE1	DOK7:S419	9.10e-9	3.12
EC	I/II	Q99569	PKP4:S406	9.13e-3	1.59
	III/IV	Q9UQ35	SRRM2:T1880	1.12e-2	1.86
MC	I/II	Q14802	FXYD3:T76	2.23e-5	3.72
	III/IV	Q14195	DPYSL3:T509	3.04e-3	2.76
CCC	I/II	Q6ZVX7	NCCRP1:S28	9.05e-8	3.65
	III/IV	Q6ZVX7	NCCRP1:S28	9.84e-9	2.75
Downregulated					
HGSC	I/II	Q9H7D0	DOCK5:S1789	8.35e-3	-1.87
	III/IV	Q8WX93	PALLD:S1104	2.21e-2	-2.01
EC	I/II	Q8TE68	EPS8L1:T187	3.11e-2	-1.80
	III/IV	-	-	-	-
MC	I/II	Q69YN4	VIRMA:S1579	1.77e-2	-2.62
	III/IV	Q16890	TPD52L1:S149	3.51e-4	-2.60
CCC	I/II	P29590	PML:S527	1.18e-2	-2.11
	III/IV	O15164	TRIM24:S811	3.72e-3	-2.72

The phosphosites of highest (upregulated) and lowest (downregulated) log2 FC for each histotype for both early-stage (I/II) and advanced stage (III/IV) patients after filtering for significantly dysregulated sites (FDR ≤ 0.01, | FC | ≥ 1.5) when comparing a given histotype to the others combined within each stage group. Displayed log2 FC values represent the fold change when the intensity of the histotype is compared to the others combined within the stage group. FDR is Benjamini-Hochberg corrected *p*-values from two-sided empirical Bayes moderated t-tests.

*CCC* Clear cell ovarian carcinoma, *EC* Endometrioid ovarian carcinoma, *FDR* False discovery rate, *HGSC* High-grade serous ovarian carcinoma, *MC* Mucinous ovarian carcinoma, *log 2* FC log2 fold change.

Functional analysis was performed to prioritize sites of known functional relevance as described by Ochoa et al. ^30^. Based on highest functional scores, WWTR1:S89, NUCKS1:S181, and CTNNB1:S675 were significantly differentially regulated between histotype stage groups. The most functional sites appeared to be mostly stage-dependent for each histotype (Fig. 1h, Supplementary Data 4). Analysis of external tumor–normal phosphoproteomic data in cProSite demonstrated significant tumor-associated upregulation (unpaired t-test *p* < 0.05) of all phosphosites with the highest functional scores, apart from SPTBN:S2169, in an independent setting (Supplementary Table 2; Supplementary Figs. 3, 4).

### Inferring kinase activity reveals stage- and histotype-specific patterns

**Fig. 2:**
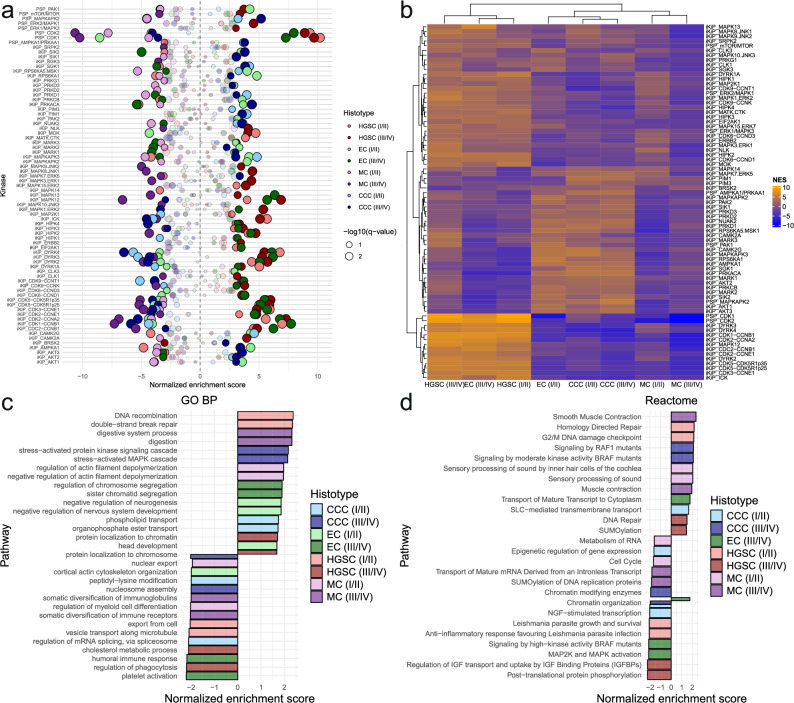
PTM-SEA and GSEA of the phosphoproteome by histotype and stage. **a** Dotplot of PTM-SEA of kinases in early stages (I/II) and advanced stages (III/IV) by histotype. Significantly enriched kinases (FDR ≤ 0.01) are indicated by opaque colors for each histotype in each stage group, whereas non-significance is transparent. **b** Heatmap and clustering of normalized enrichment scores from PTM-SEA. **c**, **d** Barplots of the top 2 significantly (FDR ≤ 0.05) up- and downregulated pathways in GSEA for parent proteins with averaged phosphosite intensities for each histotype in early- and advanced stages using the databases **c** GO BP and **d** Reactome. BP Biological process, CCC Clear cell ovarian carcinoma, EC Endometrioid ovarian carcinoma, FDR False discovery rate, GO Gene ontology, GSEA Gene set enrichment analysis, HGSC High-grade serous ovarian carcinoma, KEGG Kyoto Encyclopedia of Genes and Genomes, MC Mucinous ovarian carcinoma, PTM-SEA Post-translational modification signature enrichment analysis. FDR is based on *p*-values from the enrichment analysis from ssGSEA2.0 for PTM-SEA and clusterProfiler for GSEA, all adjusted using the Benjamini-Hochberg model. PSP and iKiP prefixes indicate kinase database origin.

Utilizing phosphosite annotations from PhosphoSitePlus (PSP) and in vitro Kinase-to-Phosphosite database (iKiP-DB), kinase activity was inferred for the phosphorylation state of the histotypes in early- and advanced stage by performing post-translational modification signature enrichment analysis. Sites regulated by CDK1 and CDK2 were highly significantly upregulated (Benjamini-Hochberg FDR ≤ 0.01) in HGSC and advanced stage EC, whereas they were distinctly downregulated in the other histotype groups, including early-stage EC (Fig. 2a). This pattern was also observed for CDK2 − CCNA2, CDK1 − CCNB1, DYRK2, DYRK3, DYRK4, and MAPK12. Clustering based on normalized enrichment scores (NES) highlighted a cluster of kinases specific for HGSC and advanced stage EC where these were included. Moreover, AKT1, AKT2, and AKT3-regulated sites were upregulated in CCC and early-stage EC, whereas downregulation was consistent in MC, early-stage CCC, and advanced stage EC. A similar activity profile was observed for kinases from the MARK, MAPK, and the PRKD-family (Fig. 2a, b; Supplementary Data 5).

### Enrichment analysis of parental proteins indicates key biological processes and signaling pathways perturbed by phosphorylation in each histotype

A gene-centric enrichment approach using curated databases from MsigDB provided additional biological insights into pathways dysregulated by phosphorylation with stage-specificity within each histotype (Supplementary Data 6)^31^. Enriched biological processes (BP) indicate unique traits between early stages and progressed disease states. Notably, significant (Benjamini-Hochberg FDR ≤ 0.20) high upregulation of DNA recombination and double-strand break repair was observed in early-stage HGSC, while protein localization-associated processes were specific to advanced stage HGSC, although the Reactome database indicated DNA repair to be a central pathway in advanced stage as well (Fig. 2c, d). In contrast, upregulation of chromosome and chromatid segregation distinguished advanced stage EC from the other histotypes (Fig. 2c). MAPK cascades, which were upregulated in CCC and downregulated in advanced stage EC in PTM-SEA, were consistent for enrichment with gene ontology (GO) BP and Reactome (Fig. 2a, c, d).

### Survival analysis suggests unique prognostic phosphosites in early- and late -stage disease by histotype

**Fig. 3:**
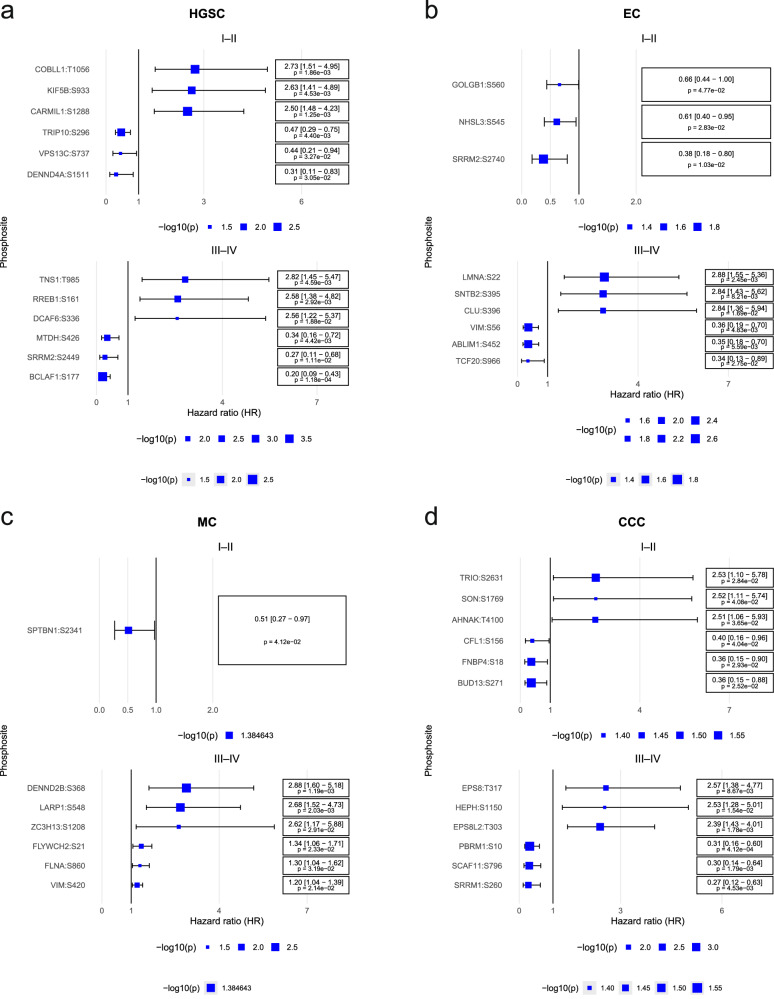
Histotype-specific phosphosites associated with clinical outcome. Forest plot of the phosphosites significantly associated (adjusted *p*-value ≤ 0.05, HR ≠ 1) with most substantial increased risk (highest HR) and decreased (lowest HR) risk of death based on overall survival using Cox proportional hazard models adjusted for age at diagnosis and stage for **a** HGSC, **b** EC, **c** MC, and **d** CCC. CCC Clear cell ovarian carcinoma, EC Endometrioid ovarian carcinoma, FDR False discovery rate, HGSC High-grade serous ovarian carcinoma, HR Hazard ratio, MC Mucinous ovarian carcinoma. Displayed *p*-values (Wald test) from Cox models have been adjusted using the Benjamini-Hochberg model.

Linking phosphosite abundance and clinical data may reveal phosphosites associated with patient outcome. Therefore, we utilized multivariate Cox proportional hazard models adjusted for age at diagnosis and stage to estimate whether there are phosphosites where higher abundances are connected to poor (Hazard ratio [HR] > 1) or favorable (HR < 1) prognosis. This analysis revealed significant (adjusted *p*-value ≤ 0.05, HR ≠ 1) prognostic phosphosites for all histotypes (Supplementary Data 7), and these were used to construct panels with sites associated with highest and poorest prognosis (Fig. 3a-d). Although histotype stage groups displayed distinct prognosic proteins, phosphorylation of SRRM-proteins appeared to generally favor improved prognosis (Fig. 3a, b, d). In contrast, two DENN domain proteins (DENND4A, DENND2B) were found to have varying prognostic implications between histotypes when phosphorylated (Fig. 3a, c). The prognostic effect of these sites was validated at protein-level using the cohort curated by Qian et al. ^32^. In total, only 20 phosphosites showed prognostic effect also at the protein-level, and these belonged to either advanced-stage HGSC or EC and did not overlap with any of the phosphosites having strongest association with favorable or unfavorable outcomes based on HR (Fig. 3a-d, Supplementary Data 8). Furthermore, 40% of these showed opposite association with survival between the datasets. The lack of results could be to the few patients for the rarer histotypes EC (I-II = 18, III-IV = 21), MC (I-II = 8, III-IV = 6), and CCC (I-II = 24, III-IV = 14) after filtering for cases with complete clinical data in the external cohort. This was however, not the case for HGSC (I-II = 54, III-IV = 465).

### Phosphosites may act as multi-purpose biomarkers

**Fig. 4:**
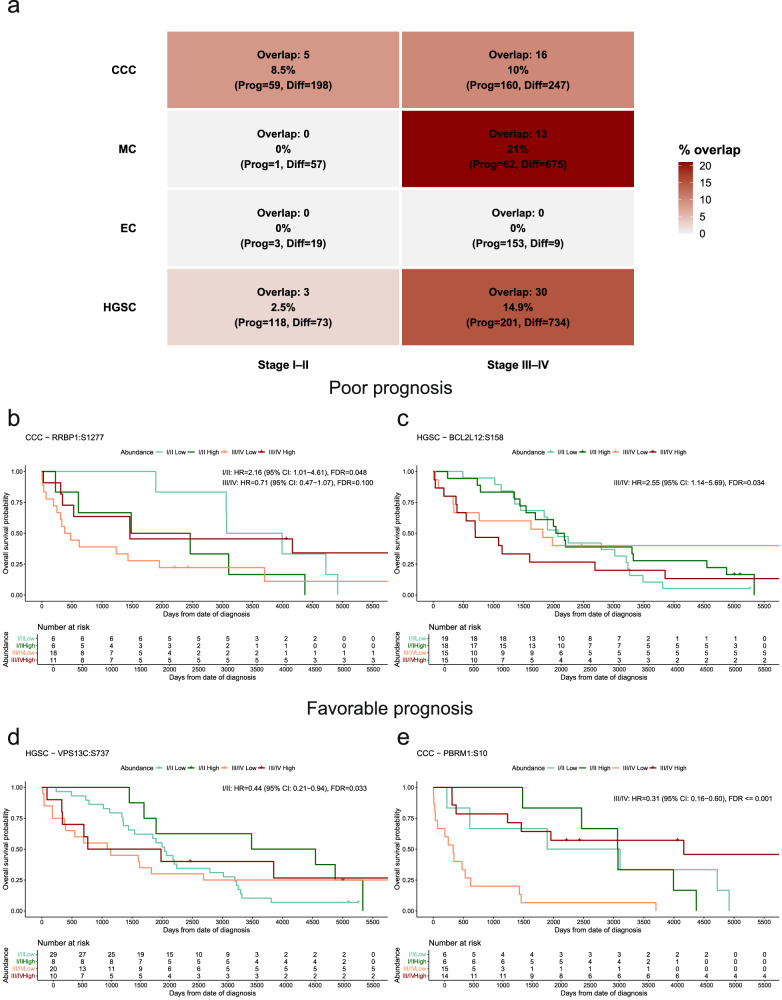
The overlap between survival and differentially abundant phosphosites. **a** Heatmap of the overlap profiles between phosphosites that are differentially abundant (FDR ≤ 0.05, | FC | ≥ 1.5) and associated with increased or decreased risk of death (FDR ≤ 0.05, HR ≠ 1) in early- and advanced stage for each histotype. Kaplan-Meier plots of phosphosites associated with the poorest clinical outcome (highest HR) in **b** early stages and **c** advanced stages. Equivalent Kaplan-Meier plots of sites connected to most favorable outcome (lowest HR) in **d** early stages and **e** advanced stages. CCC Clear cell ovarian carcinoma, EC Endometrioid ovarian carcinoma, Diff Differentially phosphorylated, FDR False discovery rate, HGSC High-grade serous ovarian carcinoma, HR Hazard ratio, MC Mucinous ovarian carcinoma, Prog Prognostic site. Displayed FDR from Cox models (Wald test p-values) have been adjusted using the Benjamini-Hochberg model. Log-rank *p*-values are global *p*-values for the four abundance groups. Stage groups were dichotomized into high and low abundance based on median abundance.

In pursuit of deriving multifunctional phosphosites as biomarkers, the overlap between differentially phosphorylated and survival-associated sites was assessed. All histotypes revealed phosphosites that were significant in both analyses except EC. For MC, only sites for advanced stage samples showed overlap (Fig. 4a). The resulting panels were composed of phosphosites associated with both favorable and unfavorable survival, where HGSC and CCC had the most prognostic sites in both stage groups (Supplementary Data 9). For poor prognosis, RRBP1:S1277 for CCC (upregulated in early stage) presented the highest HR (2.16) in early stages and showed an inverse trend in advanced stages, although not significant (Fig. 4b). BCL2L12:S158 was a highly unfavorable site observed in advanced stage HGSC, a site that was downregulated in advanced stages (Fig. 4c). In contrast, the downregulated early-stage site VPS13C:S737 in HGSC was highly favorable (HR = 0.44, Fig. 4d). The most favorable phosphosite was PBRM1:S10, which was significantly downregulated in CCC in advanced stages (Fig. 4e).

## Discussion

In this study, we investigated the phosphoproteomic landscape for patients diagnosed with four major EOC histotypes (HGSC, EC, MC, and CCC) across different stages. Our results revealed distinct regulation of phosphosites between histotypes. Differential phoshosite abundance showed upregulation of sites such as DOK7:S419 (HGSC) and NCCRP1:S28 (CCC) to be conserved across stages, while stage-specific upregulation was found in all histotypes, such as SRRM2:T1880 (EC). Functional analysis highlighted sites of high biological relevance among highly dysregulated sites. PTM-SEA highlighted histotype-dependent variation in CDK1/2 activity and stage-specific activity in MAPK signaling. Survival analysis identified prognostic sites across histotype groups, where SRRM-protein phosphorylation was associated with improved outcomes, whereas DENN domain protein phosphorylation had varying prognostic implications between histotypes.

Differentially abundant phosphosites may reveal dysregulated signaling that distinguishes histotypes across disease stages and identify potential therapeutic targets. By comparing each histotype to all others combined, we identified histotype-specific phosphorylation events. DOK7:S419 in HGSC and NCCRP1:S28 in CCC were consistently upregulated in both early and advanced disease. DOK7 suppresses proliferation, migration, and invasion through the PI3K/PTEN/AKT pathway in breast cancer, making its role in EOC worthy of further investigation^33^. Likewise, NCCRP1 is poorly characterized in ovarian cancer but has been linked to activation of non-specific cytotoxic cells and proposed as a therapeutic target in breast cancer^34,35^. The limited overlap between proteomic and phosphoproteomic differential expression, together with marked histotype-specific differences, highlights the complexity of PTM regulation in EOC. These findings suggest that phosphoproteomics can uncover signaling alterations and protein activity that are not evident from proteomics alone.

High functional scores can be indicative of phosphorylation events of high biological relevance for tumorigenesis. Our external validation using data from cProSite showed significant upregulation in tumor compared to normal tissue for nearly all highly dysregulated sites. Aberrant phosphorylation of the cell cycle regulator NUCKS1 at S181 was found and had highest functional score in early-stage HGSC and MC. CDK1 mediated phosphorylation occurs in response to DNA damage. NUCKS1 plays a role in uncontrolled proliferation and is evidenced to be overexpressed in EOC^36–38^. We also identified phosphorylation of CDK1 at Y15 as a distinct feature of HGSC in advanced stages. Given the central role of CDK1 in G2/M transition, this indicates a potential interplay between NUCKS1 and CDK1 in signaling cascades in HGSC. In contrast, CTNNB1 is known to be involved in cancer stemness and chemoresistance in MC and EC via the Wnt/β-catenin pathway^39^. Differential abundance analysis showed low levels of S675-phosphorylation of CTNNB1 in MC relative to the other histotypes in advanced stages. WWTR1:S89 and MAPK14:Y182 were distinct for CCC, with WWTR1:S89 being specific for early stages. Phosphorylation of the oncogene WWTR1 in the Hippo signaling pathway is well-known, and MAPK14 has been shown to be phosphorylated in response to chemotherapy in EOC^40,41^.

Inferring kinase activity and performing enrichment analysis can reveal regulators of phosphorylation. PTM-SEA results aligned with the strongly upregulated activity of CDK1 in HGSC as indicated in the functional analysis, and DNA damage and repair-related and cell cycle pathway-enrichment in HGSC correlates with the role of NUCKS1:S181, although mapping which phosphosites underlying this enrichment requires investigation. The results suggest similar activity of CDK1/2 for HGSC regardless of stage, but for EC this high activity was specific for advanced stages, while downregulation of both kinases was seen in early stages, which mimicked the activity seen in MC and CCC. In contrast, MARK, MAPK, and PRKD kinases had high activity in MC and CCC relative to the other histotypes, of which MAPK signaling cascades were confirmed to be upregulated in advanced stage CCC in GSEA. This is in concordance with the upregulation of MAPK14:Y182 seen in functional analysis for CCC stage groups. Considering the central role of MAPK signaling in tumorigenesis, the therapeutic utility of targeting these sites in EOC should be investigated further.

Prognostic phosphosites in the survival analysis may be indicative of signaling involved in tumor suppressive or promoting events in cancer, of which unfavorable sites could act as potential therapeutic targets. We identified SRRM proteins to be associated with protective effects in EOC histotypes, with SRRM2:S2740 and SRRM1:S260 being the most favorable in early-stage EC and advanced stage CCC, respectively. As SRRM proteins are involved in splicing, modulation of their activity may affect cell fate^42^. Previous studies have found phosphorylation of SRRM2 to be favorable in certain ovarian cancer cells. Since our findings suggest SRRM protein phosphorylation to be both differentially abundant between histotypes and prognostic, investigating their role in EOC as a target for therapies could be valuable. Additionally, phosphorylation of DENN domain proteins (DENND4A, DENND2B) showed varying prognostic associations in EOC histotypes. Similar conflicting pattern has been reported in previous studies, although high expression in EOC has demonstrated favorable outcomes^43,44^. The prognostic effect needs to be further investigated with external datasets, as our relatively small sample groups for the rare histotypes have likely limited the robustness of the Cox regression models.

External validation of prognostic effects of phosphosites on proteomics-level highlighted large disparities between omics levels, as underscored by the small number of prognostic sites that had a parental protein also shown to be prognostic, as well as non-aligning associations with patient outcomes. Because this analysis is greatly influenced by different survival data between datasets, rare histotype quantities, and analytical noise propagation between data layers, these results are inconclusive. Regardless, the small prognostic overlap highlights the need for multi-omics approaches to investigate the interplay between translation and subsequent PTM modulation to gain a more comprehensive understanding of key drivers in tumorigenesis affecting patient outcomes that could act as biomarkers suitable for targeted therapies.

This study has several limitations that should be considered when interpreting the results. Sample sizes were small for some stage groups, particularly early-stage MC (*n* = 8), which may reduce statistical power, affect *p*-values in differential expression and enrichment analyses, and increase the risk of overfitting in survival models. Although the empirical Bayes approach implemented in limma improves variance estimation and statistical power, it cannot fully compensate for small group sizes. Missing-value imputation may introduce bias, although a conservative strategy was used to minimize this risk. Potential effects of sample storage conditions and duration on phosphosite stability were not assessed. Incomplete clinical information, including treatment, comorbidities, menopausal status, and genetic background, may also have influenced phosphorylation patterns. Furthermore, bulk proteomics cannot distinguish cell type-specific effects. Validation in independent phosphoproteomic cohorts and with single-cell approaches would strengthen these findings. However, suitable public phosphoproteomic datasets for non-serous ovarian cancer were unavailable, so validation was limited to an external proteomics dataset.

In conclusion, this study characterized the phosphoproteome of EOC to derive stage-specific phosphosite signatures for each histotype and to identify biomarker candidates that can stratify the disease and potentially be used for therapeutic and prognostic purposes in a clinical setting. Our analysis identified dysregulated sites likely to be involved in tumorigenesis. These sites could be connected to key signaling pathways in the cell cycle, where CDK1/2 as well as DYRK, MARK, and MAPK proteins emerged as kinases of distinct activity across histotype stages. In survival analysis, SRRM phosphorylation was favorable and conserved across histotypes, while DENN domain protein phosphorylation displayed varying prognostic implications. Prognostic sites specific to stage groups were also identified. Collectively, these findings provide insights into signaling events characterizing early- and advanced stages in EOC histotypes. Dysregulated and prognostic phosphosites represent candidate biomarkers and therapeutic targets that warrant further experimental and clinical validation. Further validation with larger independent patient cohorts across multiple layers of omics is needed to generalize these findings as well as targeted studies investigating the therapeutic effect of identified sites and signaling pathways.

## Methods

### Patient samples

Fresh-frozen primary tumor tissues and paired formalin-fixed paraffin-embedded (FFPE) blocks from 180 patients diagnosed with EOC between 1993 and 2018 were obtained from the tumor biobank at the Sahlgrenska University Hospital, Department of Oncology (Gothenburg, Sweden). Inclusion criteria were primary tumor tissue samples originating from ovarian or fallopian tube epithelium and to be diagnosed with one of the EOC histotypes after reclassification. LGSC patients were excluded from this study as the total number of cases found was deemed insufficient for subsequent analysis. Clinicopathologic data were retrieved from the Swedish Quality Register for Gynecological Cancer National Quality Registry at the Regional Cancer Center West (Gothenburg, Sweden) and the Cancer Registry at the National Board of Health and Welfare (Stockholm, Sweden). Stage upon diagnosis was determined using the International Federation of Gynecology and Obstetrics (FIGO) guidelines, and the tumor specimens were reclassified according to the 2020 World Health Organization guidelines by a board-certified pathologist at Sahlgrenska University Hospital using the FFPE material from the same primary tumor as the fresh-frozen sample used for each patient to run on the LC-MS system. If FFPE material was not available for a patient, the frozen tissue was subjected to formalin fixation, paraffin embedding, and hematoxylin and eosin staining for reclassification. All procedures were performed in accordance with the Declaration of Helsinki and approved by the Regional Ethical Review Board (Gothenburg, Sweden; case numbers 767-14 and 201-15, and complementary case numbers T973-15 and T333-16). The Regional Ethical Review Board approved a waiver of written consent to use the tumor specimens due to the retrospective study design.

### Sample preparation

Fresh-frozen tissue pieces (8 mm^3^) containing at least 60% neoplastic cells assessed with May-Grünwald Giemsa stainings by a board-certified pathologist were homogenized in 2% sodium dodecyl sulfate and 50 mM triethylammonium bicarbonate (TEAB) using a Covaris ML230 ultrasonicator, and protein content was determined with bicinchronic acid (BCA) using the Pierce BCA Protein Assay Kit (ThermoFisher Scientific). A total of 150 µg bulk protein per sample was transferred into a 96-well KingFisher plate. The volume was adjusted to 150 µL using lysis buffer (100 mM TEAB, 2% SDS) with an Opentrons OT-2 liquid handler. In total, 150 µL of 2× reduction/alkylation buffer was added to each sample, followed by incubation at 95 °C for 15 min to ensure complete disulfide reduction and alkylation. Protein binding was performed by adding 700 µL of acetonitrile and 300 µg of MagReSyn Hydroxyl beads (Resyn Biosciences) directly to the sample plate. Protein aggregation capture (PAC) and digestion were automated on a KingFisher™ Flex system using dedicated reagent plates containing digestion enzymes (Lys-C 1:500, w/w, Thermo Fisher; and trypsin 1:250, w/w, Promega) and wash buffers. Following digestion and acidification, the samples were prepared for desalting using Oasis HLB Prime 96-well plates (Waters). Plates were conditioned with 100% acetonitrile, equilibrated with 0.1% TFA, and the samples were loaded in three times to maximize peptide retention. After washing with 0.1% TFA, peptides were eluted using 60% acetonitrile, 5% TFA, and 0.1 M glycolic acid, and collected into a PCR plate for storage at −80 °C.

### Phosphopeptide enrichment

Phosphopeptides were enriched using MagReSyn Zr-IMAC HP beads (Resyn Biosciences) on the KingFisher system. Beads were used at a mass ratio of at least 2:1 (beads:peptide). Peptides were mixed with binding solvent containing 100% acetonitrile, 5% TFA, and 0.1 M glycolic acid. Wash steps included two buffers: Wash 1 (80% acetonitrile, 1% TFA) and Wash 2 (10% acetonitrile, 0.2% TFA). Elution was performed with 1% aqueous ammonia (pH 10–11), and eluates were immediately frozen at −80 °C.

### Liquid chromatography-mass spectrometry and identification

Phosphopeptides were analyzed on an Evosep One LC system (Whisper zoom mode 40SPD, 32-minute method) coupled to a Thermo Q Exactive HF-X Orbitrap mass spectrometer via electrospray ionization in a randomized order. Data were acquired in data-independent acquisition (DIA) mode using Xcalibur software. Full MS scans were acquired at a resolution of 120,000 over an m/z range of 350–1400, with an AGC target of 3 × 10⁶ and a maximum injection time of 25 ms. MS2 scans were recorded at 15,000 resolution using 50 staggered DIA windows of 13.7 m/z, with normalized collision energy (NCE) set to 27. Fragmentation spectra were acquired in centroid mode under positive ionization. Identification was done in DIA-NN (2.2.0). With randomized pooled samples, an in-silico spectral library was first generated using a reviewed UniProt database (Swissprot, February 2025, 20,504 entries) with deep learning-based spectra, RTs and Ims prediction and contaminants enabled and generic scoring, and set to include contaminants. Library searching was conducted with Trypsin/P as protease with maximum one missed cleavage, and N-terminal methionine excision was enabled and cysteine carbamidomethylation set as fixed modification. STY was set as variable modifications maximum number of variable modifications set to 2. Sample intensities were normalized with RT-dependent cross-run normalization and match between runs (MBR) was enabled to adjust for sample-to-sample variations and batch effects.

### Data pre-processing

All subsequent analyses were conducted using R (v. 4.5.1) unless otherwise stated. Site-centric data were generated from the parquet files generated by DIA-NN. Phosphosites were filtered for ≥ 0.75 localization probability score. A filtering threshold of 0.01 was applied for global q-value, peptidoform q-value, and global peptidoform q-value. Filtered phosphosites were merged with intensities for each precursor mapping to the same site. To generate one intensity for each unique phosphosite, aggregation of precursors was done by averaging their normalized intensity scores for each site. Phopshosites were filtered for quantitative values in at least 30% of samples in at least one histotype-stage group. Remaining missing values were imputed by replacing them with the lowest detected intensity across all samples for respective phosphosite, and then log2(n + 1)-transformed.

### Differential abundance analysis

Differentially phosphorylated sites were generated using NormalyzerDE (v. 1.26.0) with the limma method with minimum number of replicates set to 3, and Benjamini-Hochberg to acquire FDR-adjusted *p*-values. Abundances between histotypes were examined by contrasting one histotype to the others combined for early- and advanced stage samples separately. A site was deemed significantly deregulated at thresholds FDR ≤ 0.05 and FC ≥ |1.5 | . The *p*-values were obtained from two-sided empirical Bayes moderated t-tests. This analysis was repeated using the proteomics data. An additional differential abundance analysis was performed where the phosphosite intensities were adjusted by protein levels using proteomics data from a previous experiment using lysates from the same patients. Here, the phosphosite intensity was divided by the intensity of the corresponding protein for each sample. The proteomics data were searched with DIA-NN (2.2.0) using the same FASTA library for in-silico library generation and normalization settings as for the phosphoproteomics data, except for setting no variable modifications for proteomics data.

### Enrichment analysis

Gene set enrichment analysis (GSEA) was performed by taking the average intensities of all phosphosites mapping to the same gene for each sample. Using clusterProfiler (v. 4.16.0), enrichment of the gene ontology (GO) biological processes (BP) and Reactome databases from MsigDB were conducted on genes ranked from highest to lowest ratio of the following formula^31^:

$$-\log 10({\rm{FDR}})/{\rm{sign}}(\log 2{\rm{FC}})$$

where FDR and log2 FC were derived from the differential abundance analysis. The sign of log2 FC is defined as 1 if above 0, and -1 if below 0. Significance threshold for enrichment was Benjamini-Hochberg adjusted *p*-value < 0.20.

Post-translational modification signature enrichment analysis (PTM-SEA) was done with ssGSEA (v. 1.0.0) using the PTM signature database (ptm.sig.db.KINASE.flanking.human.v2.0.0.gmt) curated at Broad Institute, which incorporates PhosphoSitePlus (PSP) and in vitro kinase-to-phosphosite database (iKiP-DB)^22^. The enrichment was performed setting weight as 0.75, correlation type as rank, “area.under.RES” as statistic, number of permutations 500, and global FDR to false. Significance thresholds were set to FDR < 0.01 and a minimum signature set overlap of 10%.

### Functional analysis

Predicting the functional scores of phosphosites was done by employing the machine learning pipeline established by Ochoa et al., where all identified phosphosites with a localization probability ≥ 0.75 from this study were used as reference and funscoR (v. 0.1.0) for model training and functional score prediction for S, T, and Y phosphosites^30^.

### Validation with external data

Comparison of phosphosite in abundance in tumor and normal tissue was done by utilizing publicly available data on The Cancer Proteogenomic Data Analysis Site (cProSite), curated by National Cancer Institute’s Clinical Proteomic Tumor Analysis Consortium (CPTAC) and National Cancer Institute’s International Cancer Proteogenome Consortium (ICPC)^45^. Their web-based platform was used to search for comparisons of phosphosites between tumor and adjacent normal tissue in ovarian cancer by setting the chosen tumor type as ovarian cancer, dataset as phosphorylation site, analysis as tumor vs normal tissue, and gene as the given phosphorylated protein. Unpaired t-tests, fold changes, and boxplots were then extracted after choosing the phosphosite to be validated. This was performed for the three phosphosites of highest functional score in each histotype and stage group.

### Survival analysis

Multivariate Cox regression for overall survival was performed for each histotype across both stage groups by generating Cox proportional hazard (PH) models adjusted for age at diagnosis and stage using the coxph-function in survival (v. 3.8-3). From the models, hazard ratio (HR), confidence interval (CI) for HR, FDR (Benjamini-Hochberg adjusted), and concordance index (C-index) were obtained. Overall survival was defined as the time between date of diagnosis and death by any cause. The *p*-values were acquired using Wald tests. This was performed for all phosphosites post-filtering, where Cox PH models were generated for all sites and covariates for those selected using least absolute shrinkage and selection operator (LASSO) using glmnet (v. 4.1-10) with alpha = 1. Robustness of survival models was assessed by bootstrapping the survival data for 1000 iterations, generating a bootstrap-adjusted *p*-value. This analysis was repeated using clinical data and proteomics data published by Qian et al. ^32^ for progression-free survival analysis, where the date of relapse was defined as the event. *P*-values from log-rank tests were estimated for all phosphosites significantly associated with survival based on the Cox PH models (FDR ≤ 0.05, bootstrap *p*-value ≤ 0.20, HR ≠ 1 and CI not spanning 1), where log2-tranformed intensities were dichotomized based on median abundance for the specific histotype stage-group. This methodology was used to construct Kaplan-Meier plots.

### Visualizations

Principal component analysis (PCA) plots, volcano plots, barplots, dotplots, and Kaplan-Meier curves were custom designed using ggplot2 (v. 3.5.2). Heatmaps were constructed with ComplexHeatmap (v. 2.24.1) using Euclidean distance for unsupervised clustering.

## Supplementary information

Supplementary information

Supplementary Data

## Data Availability

The mass spectrometry phosphoproteomics data and metadata have been deposited to the ProteomeXchange Consortium via the PRIDE^46^ partner repository with the dataset identifier PXD069845, and the proteomics data are available at the MassIVE repository with the dataset identifier MSV000097588.

## Code availability

All custom R scripts are publicly available at https://github.com/lwernerGU/Phosphoproteomics-analysis-pipeline.
