# Supplementary material for: The phosphoproteome of ovarian carcinoma delineates signaling signatures and potentially druggable targets across histotype stages
Source: NPJ Precis Oncol. 2026 Aug 14;10:317. doi: 10.1038/s41698-026-01659-9 (PMC13476440; doi:10.1038/s41698-026-01659-9)
Supplement: Supplementary file 1 [file 41698_2026_1659_MOESM1_ESM.pdf]

Supplementary Tables

Supplementary Table 1. Phosphosites of highest differential abundance for each histotype after adjusting for parent protein levels.

The phosphosites of highest (upregulated) and lowest (downregulated) log2 FC for each histotype for both early-stage (I/II) and advanced stage (III/IV) patients after filtering for significantly dysregulated sites (FDR ≤ 0.01, | FC | ≥ 1.5) for phosphosite intensities adjusted by parental protein abundance from a paired proteomics data set. CCC Clear cell ovarian carcinoma, EC Endometrioid ovarian carcinoma, FDR False discovery rate, HGSC High-grade serous ovarian carcinoma, MC Mucinous ovarian carcinoma, log 2 FC log2 fold change. Displayed log2 FC values represent the fold change when the intensity of the histotype is compared to the others combined within the stage group. FDR is Benjamini-Hochberg corrected p-values from two-sided empirical Bayes moderated t-tests.

**Upregulated**					
**Histotype**	**Stage**	**Protein accession**	**Phosphosite**	**FDR**	**Log2 FC**
HGSC	I/II	Q9H1E3	NUCKS1:S181	8.34e-3	1.20
	III/IV	P19338	NCL:S67	4.07e-2	1.48
EC	I/II	-	-	-	-
	III/IV	-	-	-	-
MC	I/II	P20700	LMNB1:S391	3.65e-2	1.16
	III/IV	O60716	CTNND1:S920	2.23-2	0.95
CCC	I/II	-	-	-	-
	III/IV	O14578	CIT:S480	4.32e-2	2.41
**Downregulated**					
HGSC	I/II	Q9UPX8	SHANK2:S1380	3.89e-2	-2.55
	III/IV	P55196	AFDN:S1721	4.90e-2	-1.67
EC	I/II	Q9H501	ESF1:S153	1.00e-2	-1.17
	III/IV	-	-	-	-
MC	I/II	-	-	-	-
	III/IV	Q8N4C8	MINK1:S701	2.56e-2	-1.95
CCC	I/II	-	-	-	-
	III/IV	Q96JM7	L3MBTL3:S608	2.19e-2	-0.69

Supplementary Table 2. Phosphosite level compared to normal tissue for sites of highest functional score.

P-values and log2 FC from the Cancer Proteogenomic Data Analysis Site (cProSite), curated by National Cancer Institute's Clinical Proteomic Tumor Analysis Consortium (CPTAC) and National Cancer Institute's International Cancer Proteogenome Consortium (ICPC), for the phosphosites of highest functional scores in this study. Reported p-values and log2 FC are based on unpaired t-tests where abundance in tumor tissue was compared

to adjacent normal tissue. If the phosphosite was not found in cProSite, no data was reported. CCC Clear cell ovarian carcinoma, EC Endometrioid ovarian carcinoma, HGSC High-grade serous ovarian carcinoma, log 2 FC log2 fold change, MC Mucinous ovarian carcinoma.

Phosphosite	Histotype(s)	Stage	Functional score	p-value	Average log2 FC (tumor/normal)
WWTR1:S89	HGSC	III/IV	0.99	No data	No data
	CCC	I/II			
ADAM22:S834	CCC	I/II	0.96	No data	No data
MAPK14:Y182	CCC	I/II	0.96	< 0.0001	1.45
	CCC	III/IV			
ACLY:S455	CCC	III/IV	0.95	0.0352	1.10
PML:S527	CCC	III/IV	0.94	< 0.0001	1.43
CARHSP1:S41	EC	I/II	0.97	0.0006	0.97
WHRN:S245	EC	I/II	0.76	0.0070	1.05
PLEC:S4396	EC	I/II	0.71	0.0058	0.85
GATAD2A:S107	EC	III/IV	0.65	No data	No data
HNF1A:S247	EC	III/IV	0.64	No data	No data
DENR:S73	EC	III/IV	0.62	0.0001	1.46
NUCKS1:S181	HGSC	I/II	0.95	< 0.0001	2.27
	MC	I/II			
FLNA:S1084	HGSC	I/II	0.94	0.0002	1.15
RPL12:S38	HGSC	I/II	0.91	< 0.0001	2.06
CDK1:Y15	HGSC	III/IV	0.99	< 0.0001	2.59
CTNNB1:S675	HGSC	III/IV	0.98	0.0013	1.91
	MC	III/IV			
TMC5:S79	MC	I/II	0.88	0.0289	2.95
SPTBN1:S2169	MC	I/II	0.87	0.2170	0.61
OCIAD1:S108	MC	III/IV	0.99	< 0.0001	1.88
PDPK1:S241	MC	III/IV	0.98	0.0035	0.67

Supplementary Figures

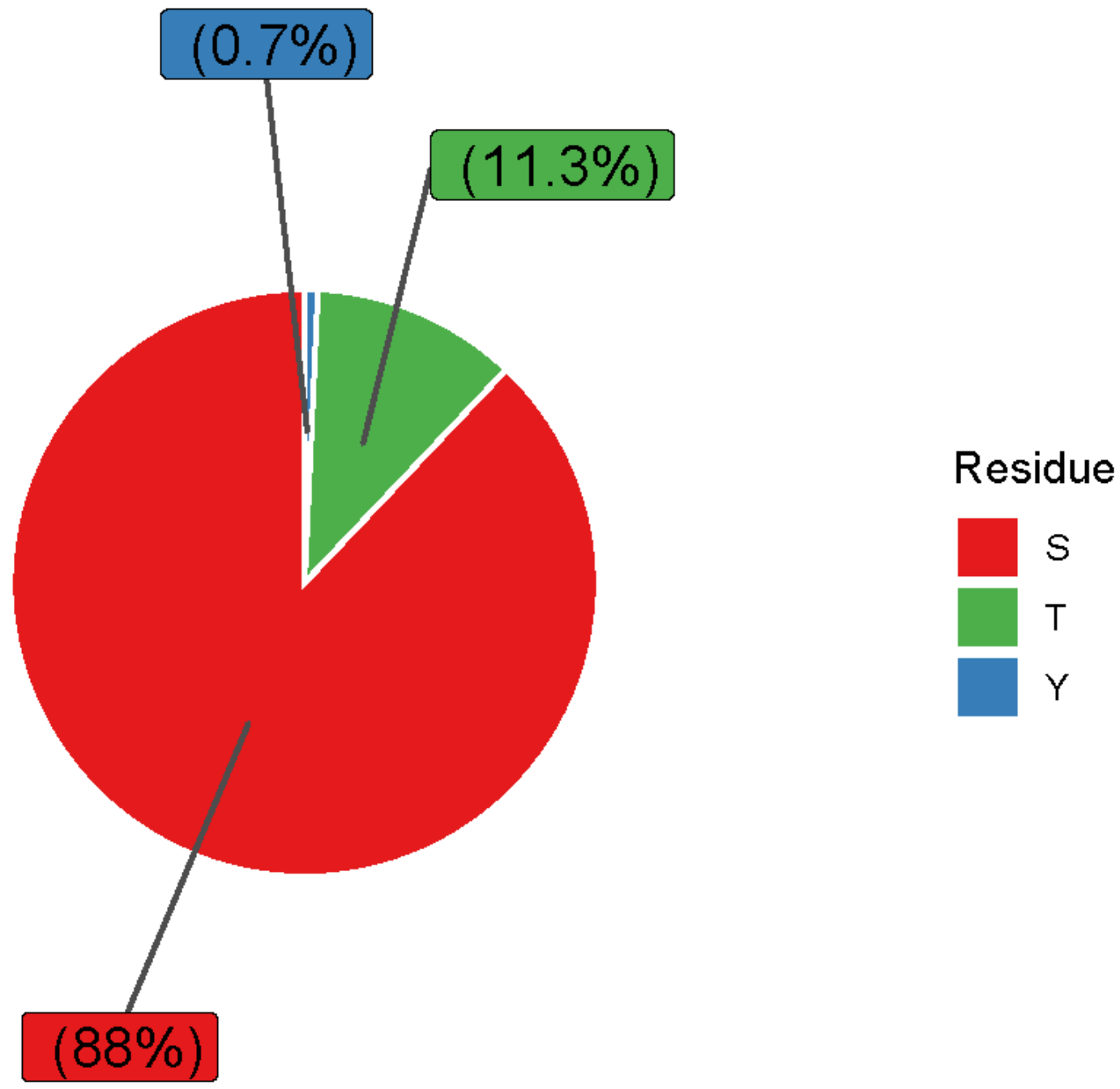

Supplementary Figure 1. Distribution of phosphorylated residues.

Pie chart of the relative percentage of amino acids phosphorylated among all confidently localized (≥ 0.75) sites prior to downstream phosphosite filtering. S Serine, T Threonine, Y Tyrosine.

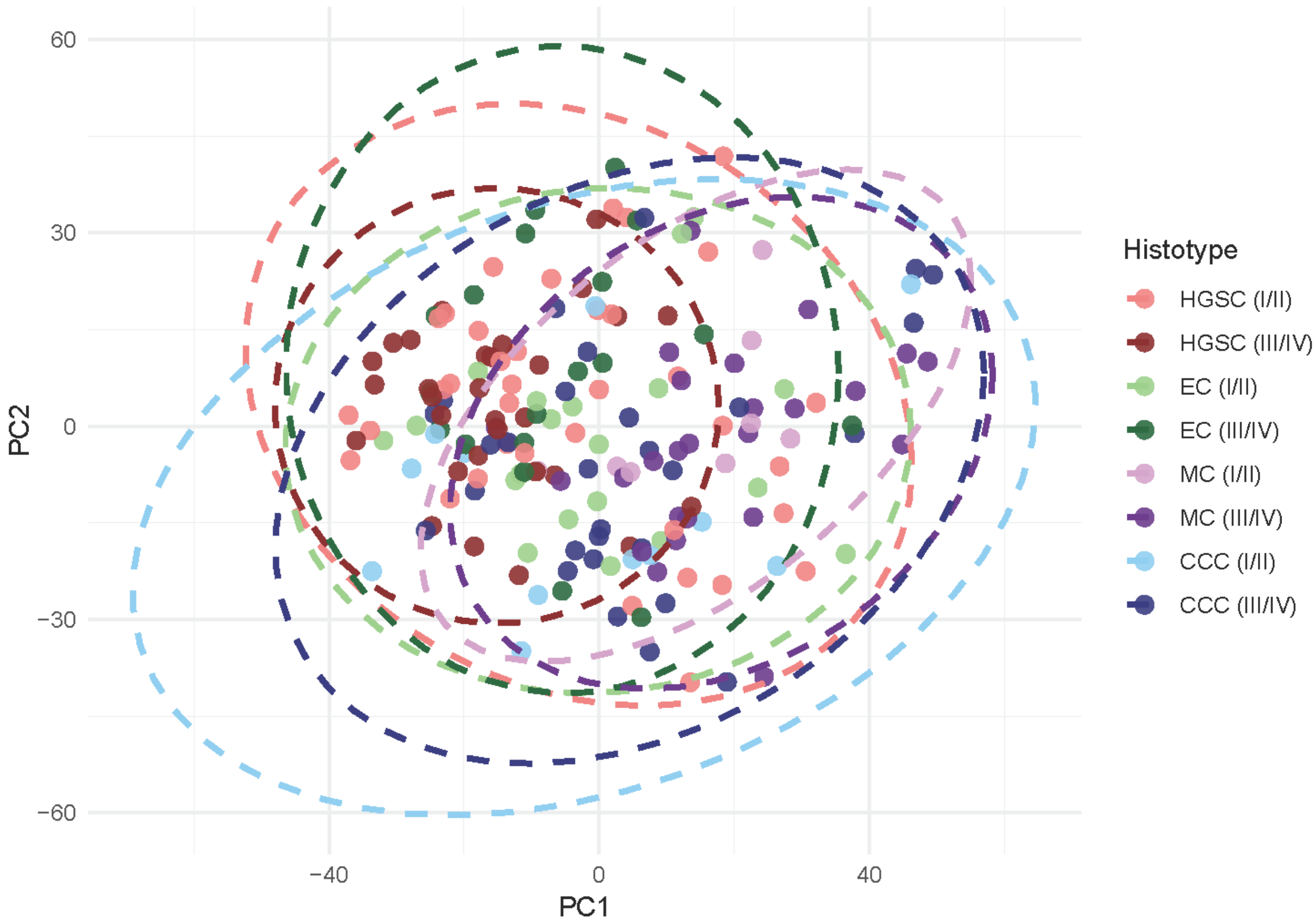

Supplementary Figure 2. PCA plot of phosphosite abundances for each histotype in early- and advanced stages.

A scatter plot of the data distribution from PCA of log2-transformed phosphosite intensities for early-stage (I/II) and advanced stage (III/IV) samples for all histotypes. CCC Clear cell ovarian carcinoma, EC Endometrioid ovarian carcinoma, HGSC High-grade serous ovarian carcinoma, MC Mucinous ovarian carcinoma. Missing data were replaced with 0.

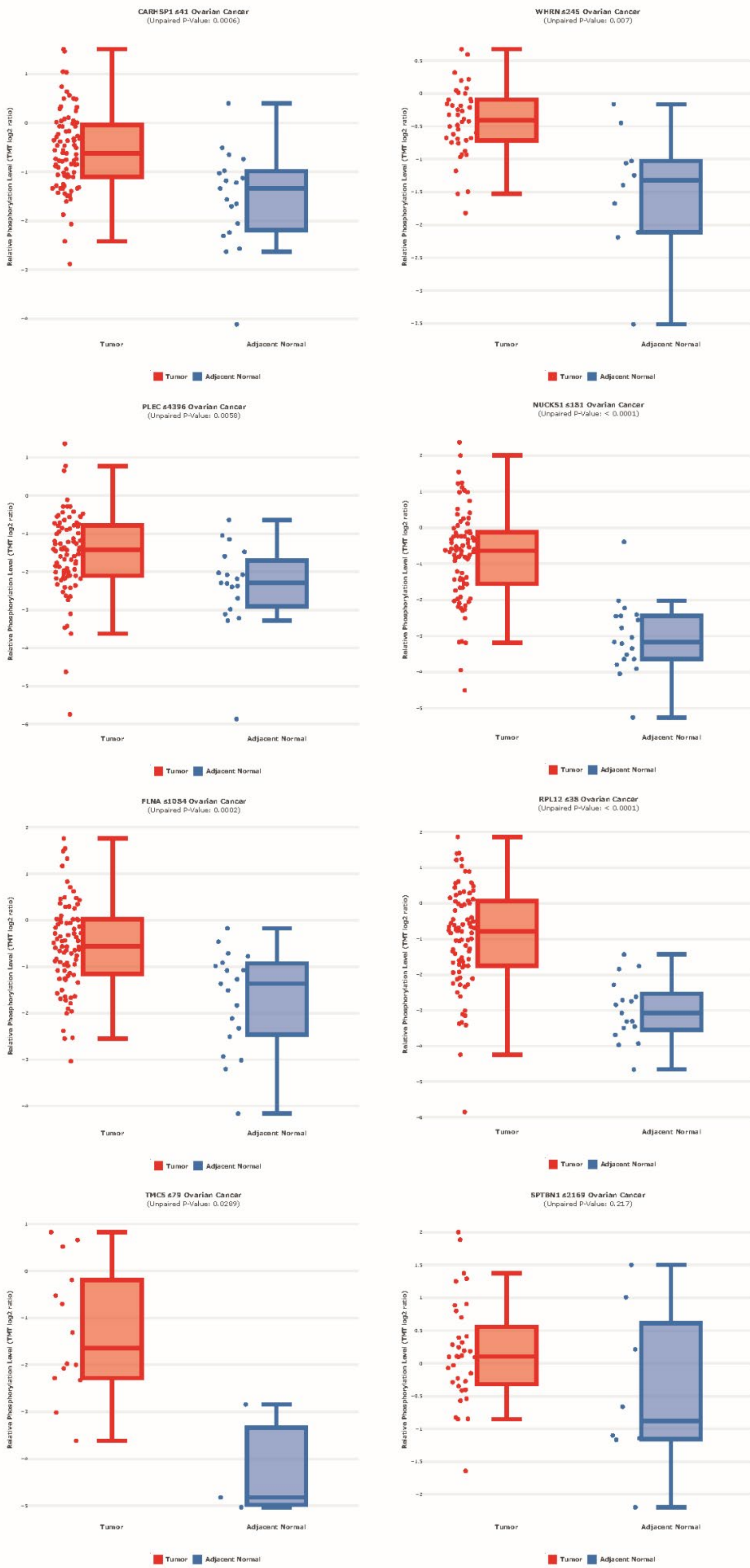
CARHSP1 s41 Ovarian Cancer
(Unpaired P-Value: 0.0006)
WHRN s245 Ovarian Cancer
(Unpaired P-Value: 0.007)
PLEC s4396 Ovarian Cancer
(Unpaired P-Value: 0.0058)
NUCKS1 s181 Ovarian Cancer
(Unpaired P-Value: < 0.0001)
FLNA s1084 Ovarian Cancer
(Unpaired P-Value: 0.0002)
RPL12 s38 Ovarian Cancer
(Unpaired P-Value: < 0.0001)
TMC5 s79 Ovarian Cancer
(Unpaired P-Value: 0.0289)
SPTBN1 s2169 Ovarian Cancer
(Unpaired P-Value: 0.217)
Relative Phosphorylation Level (TMT log2 ratio)
Tumor
Adjacent Normal

Supplementary Figure 3. Boxplots of phosphosite level compared to normal tissue for sites of highest functional score among in early stages.

Boxplots generated from the Cancer Proteogenomic Data Analysis Site (cProSite), curated by National Cancer Institute's Clinical Proteomic Tumor Analysis Consortium (CPTAC) and National Cancer Institute's International Cancer Proteogenome Consortium (ICPC), for the phosphosites of highest functional scores among differentially abundant site in early stages in this study. Reported p-values are based on unpaired t-tests where abundance in tumor tissue was compared to adjacent normal tissue. CCC Clear cell ovarian carcinoma, EC Endometrioid ovarian carcinoma, HGSC High-grade serous ovarian carcinoma, MC Mucinous ovarian carcinoma, TMT Tandem mass tag.

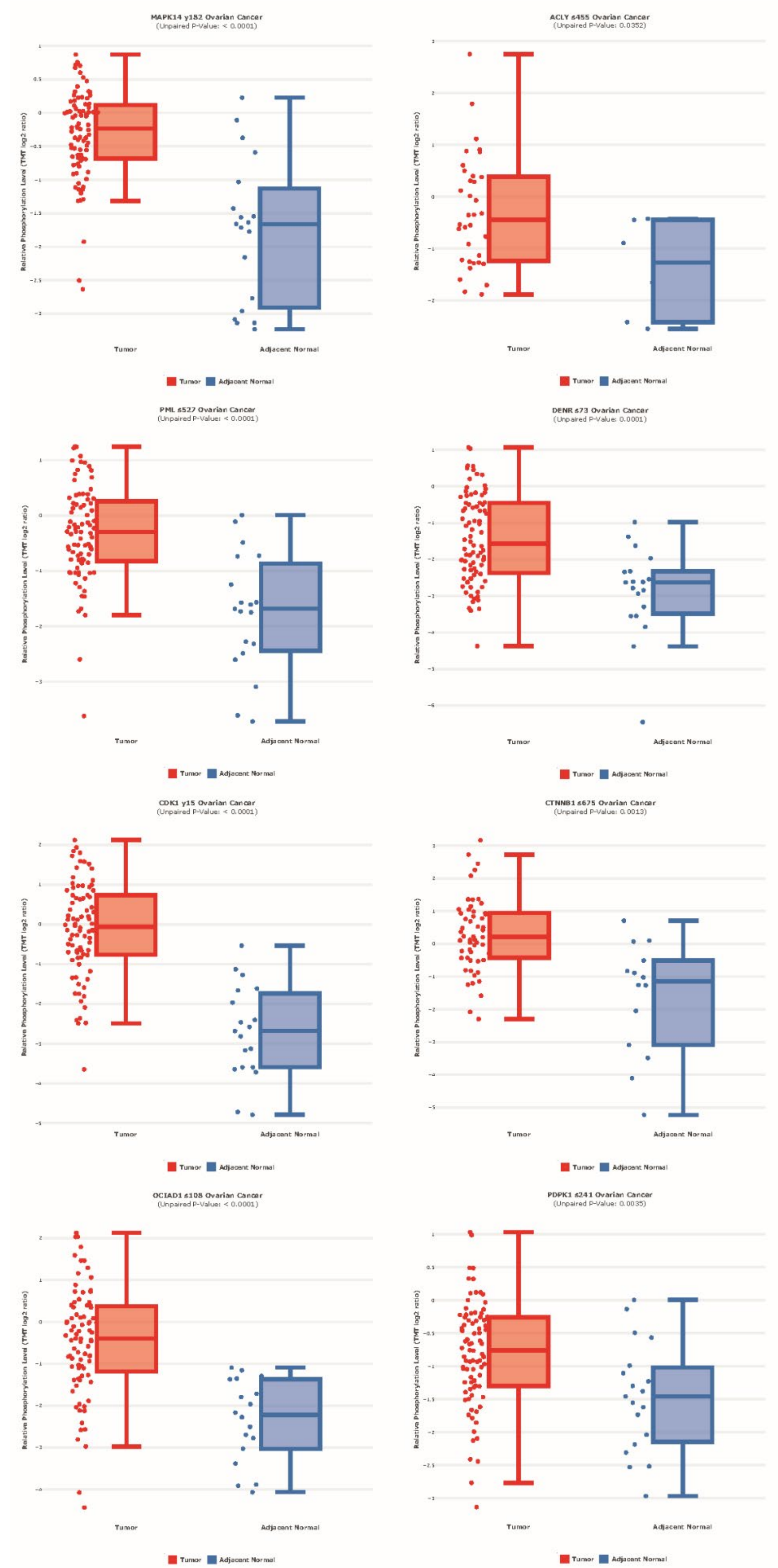
MAPK14 y182 Ovarian Cancer
(Unpaired P-Value: < 0.0001)
Relative Phosphorylation Level (TMT log2 ratio)
Tumor
Adjacent Normal
Tumor Adjacent Normal
ACLY s455 Ovarian Cancer
(Unpaired P-Value: 0.0352)
Relative Phosphorylation Level (TMT log2 ratio)
Tumor
Adjacent Normal
Tumor Adjacent Normal
PML s527 Ovarian Cancer
(Unpaired P-Value: < 0.0001)
Relative Phosphorylation Level (TMT log2 ratio)
Tumor
Adjacent Normal
Tumor Adjacent Normal
DENR s73 Ovarian Cancer
(Unpaired P-Value: 0.0001)
Relative Phosphorylation Level (TMT log2 ratio)
Tumor
Adjacent Normal
Tumor Adjacent Normal
CDK1 y15 Ovarian Cancer
(Unpaired P-Value: < 0.0001)
Relative Phosphorylation Level (TMT log2 ratio)
Tumor
Adjacent Normal
Tumor Adjacent Normal
CTNNB1 s675 Ovarian Cancer
(Unpaired P-Value: 0.0013)
Relative Phosphorylation Level (TMT log2 ratio)
Tumor
Adjacent Normal
Tumor Adjacent Normal
OCIAD1 s108 Ovarian Cancer
(Unpaired P-Value: < 0.0001)
Relative Phosphorylation Level (TMT log2 ratio)
Tumor
Adjacent Normal
Tumor Adjacent Normal
PDPK1 s241 Ovarian Cancer
(Unpaired P-Value: 0.0035)
Relative Phosphorylation Level (TMT log2 ratio)
Tumor
Adjacent Normal
Tumor Adjacent Normal

Supplementary Figure 4. Boxplots of phosphosite level compared to normal tissue for sites of highest functional score among in advanced stages.

Boxplots generated from the Cancer Proteogenomic Data Analysis Site (cProSite), curated by National Cancer Institute's Clinical Proteomic Tumor Analysis Consortium (CPTAC) and National Cancer Institute's International Cancer Proteogenome Consortium (ICPC), for the phosphosites of highest functional scores among differentially abundant site in advanced stages in this study. Reported p-values are based on unpaired t-tests where abundance in tumor tissue was compared to adjacent normal tissue. CCC Clear cell ovarian carcinoma, EC Endometrioid ovarian carcinoma, HGSC High-grade serous ovarian carcinoma, MC Mucinous ovarian carcinoma, TMT Tandem mass tag.

Supplementary Data

Data 1. Significantly differentially abundant phosphosites (FDR <= 0.05, | FC | >= 1.5) for all histotypes in early- and advanced stages, ordered from highest to lowest log2 FC within each histotype stage group.

Data 2. Significantly differentially abundant phosphosites (FDR <= 0.05, | FC | >= 1.5) for all histotypes in early- and advanced stages when phosphosite intensities have been adjusted by parent protein intensity from a paired proteomics data set prior to analysis, ordered from highest to lowest log2 FC within each histotype stage group.

Data 3. Significance overlap between differential abundance analysis results using phosphoproteome and proteome.

Data 4. Functional analysis results for all identified phosphosites.

Data 5. Kinases significantly enriched (FDR <= 0.01) in at least one histotype stage group in PTM-SEA.

Data 6. Significantly enriched (adjusted p-value <= 0.20) pathways for histotypes in early- and advanced stages using multiple databases.

Data 7. Phosphosites significantly associated with increased or decreased risk of death (FDR ≤ 0.05, bootstrap p-value ≤ 0.20, HR ≠ 1 and CI not spanning 1) for each histotype in both stage groups.

Data 8. Overlapping prognostic proteins and phosphosites for external validation on protein-level.

Data 9. Results from differential abundance analysis and survival analysis for phosphosites significant in both analyses.
